# Meiotic and mitotic behaviour of a ring/deleted chromosome 22 in human embryos determined by preimplantation genetic diagnosis for a maternal carrier

**DOI:** 10.1186/1755-8166-2-3

**Published:** 2009-01-23

**Authors:** Anna Mantzouratou, Anastasia Mania, Marianna Apergi, Sarah Laver, Paul Serhal, JDA Delhanty

**Affiliations:** 1 UCL Centre for PGD, Institute for Women's Health, University College London, 86-96 Chenies Mews, London, WC1E-6HX, UK; 2 The Assisted Conception Unit, University College Hospital, Eastman Dental Hospital, Gray's Inn Road, London, WC1X 8LD, UK

## Abstract

**Background:**

Ring chromosomes are normally associated with developmental anomalies and are rarely inherited. An exception to this rule is provided by deletion/ring cases. We were provided with a unique opportunity to investigate the meiotic segregation at oogenesis in a woman who is a carrier of a deleted/ring 22 chromosome. The couple requested preimplantation genetic diagnosis (PGD) following the birth of a son with a mosaic karyotype.

The couple underwent two cycles of PGD. Studies were performed on lymphocytes, single embryonic cells removed from 3 day-old embryos and un-transferred embryos. Analysis was carried out using fluorescence in situ hybridisation (FISH) with specific probe sets in two rounds of hybridization.

**Results:**

In total, 12 embryos were biopsied, and follow up information was obtained for 10 embryos. No embryos were completely normal or balanced for chromosome 22 by day 5. There was only one embryo diagnosed as balanced of 12 biopsied but that accumulated postzygotic errors by day 5. Three oocytes apparently had a balanced chromosome 22 complement but all had the deleted and the ring 22 and not the intact chromosome 22. After fertilisation all the embryos accumulated postzygotic errors for chromosome 22.

**Conclusion:**

The study of the preimplantation embryos in this case provided a rare and significant chance to study and understand the phenomena associated with this unusual type of anomaly during meiosis and in the earliest stages of development. It is the first reported PGD attempt for a ring chromosome abnormality.

## Background

In humans, ring chromosomes are normally associated with developmental anomalies and are rarely inherited. An exception to this rule is provided by del/ring cases where euchromatic material carried by the ring has been derived by an interstitial deletion, with one of the breaks occurring through the centromere. Ring/del cases can be considered to form a special subgroup among the small supernumerary marker chromosomes (sSMC); these are additional, abnormal chromosomes the origin of which cannot usually be determined by conventional techniques. If the additional genetic material is of euchromatic origin the sSMC may be associated with developmental anomalies, but in the case of the ring/del situation the additional material is compensated for by the deletion and the phenotype is normal.

Several examples of the ring/deleted type of anomaly are known from the literature [1-10]. The rearrangement creates a balanced carrier status, with the potential to produce abnormal offspring with a variety of unbalanced karyotypes, either duplicated or deleted for the region involved [1,4,6,7]. Prenatal diagnosis may be offered to known del/ring carriers but risk calculations will be difficult since the meiotic behaviour of this type of anomaly is unknown in humans.

We were provided with a unique opportunity to investigate the meiotic segregation at oogenesis in a woman who is a carrier of a del/ring 22 chromosome. The couple requested preimplantation genetic diagnosis (PGD) following the birth of a son with a mosaic karyotype. In his lymphocytes, one cell line had a copy of the ring 22 chromosome in addition to the normal 46,XY complement while in other cells the ring had been lost. A subsequent female pregnancy with the same karyotype was terminated. The first pregnancy had followed a period of infertility.

For PGD, the couple involved has to undergo in vitro fertilization (IVF) to enable the simultaneous testing of several embryos. One or two cells from each embryo is removed on day 3 of development and tested for the particular chromosome(s) involved to allow selection of those that are unaffected [11]. After two PGD treatment cycles and two natural pregnancies, information was available on 12 meioses, none of which produced an oocyte with a single intact chromosome 22. Three meioses resulted in balanced oocytes carrying both the deleted chromosome 22 and the ring, as in the mother. Due to the known instability of small ring chromosomes, as evidenced both in this family and others in the literature [7,12,13] this situation created a dilemma when detected at embryo biopsy. The follow up studies carried out on the embryos that were not transferred after diagnosis provided an opportunity to monitor the mitotic behaviour of the ring 22 chromosome.

## Results

**Figure 1:**
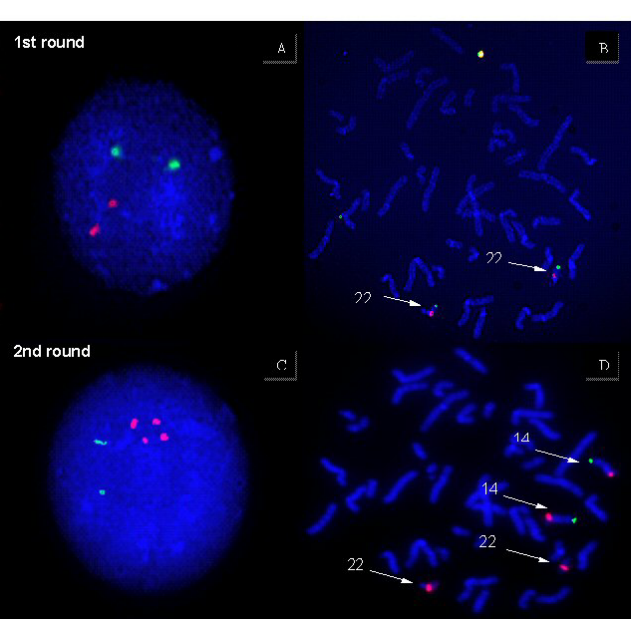
**Hybridization of diagnostic probe sets to lymphocyte nuclei of the father**. In the first round, the Di George dual band probe set is used (22q11.2 orange/22q13.3 green). In the second round the probes to detect the centromere 14/22 red and 14qtel in green are used.

**Figure 2:**
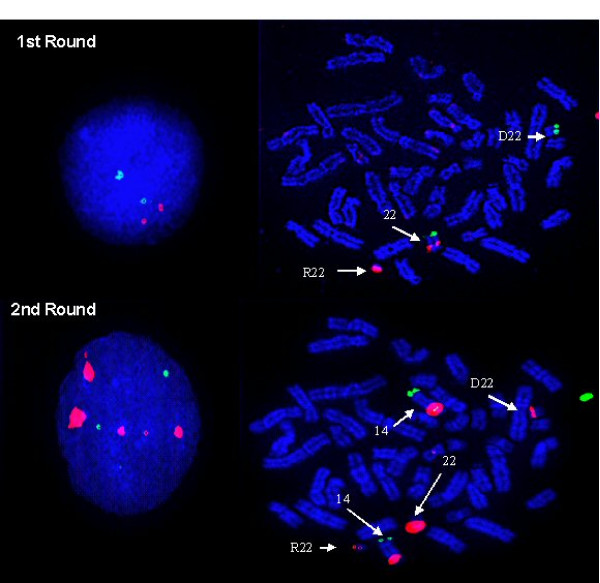
**Hybridization of diagnostic probe sets to lymphocytes of the carrier mother**. In the first round, the Di George dual band probe set is used (22q11.2 orange/22q13.3 green). In the second round the probes to detect the centromere 14/22 red and 14qtel in green are used.

**Figure 3:**
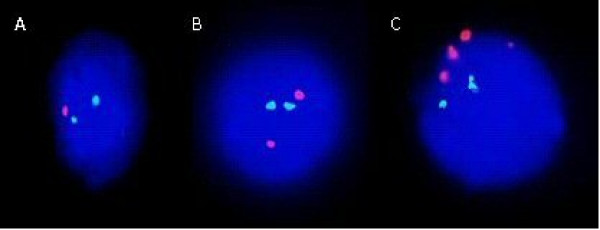
**Results of FISH analysis on embryonic nuclei with probes for the detection of the intact chromosome 22 and the ring/deleted 22 chromosome during PGD**. A. First round FISH result with the Di George dual band probe set (22q11.2 orange/22q13.3 green).on a single blastomere showing loss of ring chromosome 22 detected by the orange probe. B. Normal FISH signals for chromosome 22 with the Di George dual band probe set (22q11.2 orange/22q13.3 green)., the embryo could have two intact chromosomes 22 or be a carrier. C. Second round FISH result with probes Cep14/22 (red) and 14qtel (green) on the same cell as in B, shows a balanced carrier of the ring and deleted 22.

When considering the appropriate probes for this unusual abnormality it was decided that the balanced carriers of the maternal rearrangement needed to be detected and distinguished from embryos that had two normal copies of chromosome 22 due to the high risk of instability leading to mosaicism that is associated with the ring/del type of abnormality [7,12,13]. Cytogenetic workup in parental lymphocytes with commercially available probes for chromosome 22 showed that the centromere of chromosome 22 was split between both derivative chromosomes 22 (the deleted chromosome and the ring) in the mother. Consequently, two rounds of FISH were used in order to detect all the unbalanced and balanced carriers in the resulting embryos. The combined FISH probe efficiency on control lymphocytes was 90% and on patient lymphocytes was 95%. Figures 1 and 2 show the FISH results on control and patient lymphocytes for both metaphase and interphase nuclei. Figure 3 shows an example of the FISH results on embryonic nuclei from biopsied and untransferred embryos which are all in the interphase stage.

The couple underwent two cycles of preimplantation genetic diagnosis for the rare ring/del chromosome abnormality. There was no embryo transfer in either cycle due to all the embryos being either affected with a chromosome 22 imbalance or balanced carriers of the maternal chromosomal abnormality. Table 1 (Additonal File 1) summarises the results of the first PGD cycle. In brief, 7 oocytes were collected and 6 of them were fertilised by IVF. Five embryos were biopsied on day 3 and two cells were taken from all embryos. Unfortunately, no embryos were available for transfer. An embryo that was a balanced carrier of the ring/del chromosome was found but due to the likely instability of the ring 22 during cell division (as evidenced by cytogenetic analysis of tissues from the couple's two natural pregnancies), the couple decided not to have it transferred. All biopsied embryos were spread on slides on day 5 for full follow up analysis. Four out five embryos grew well and had reached the expected blastocyst stage. Results were obtained on follow up for four out of the five embryos (Table 2 - Additional File 1). In three embryos the meiotic imbalance detected at biopsy was confirmed but they were also aneuploid mosaics due to additional post-zygotic errors. Embryo number 2 that was diagnosed as a balanced ring/del carrier on day 3 had become mosaic abnormal by day 5 with loss of the ring in 50% of the cells. The meiotic segregation patterns of the recombinant and normal 22 chromosomes in the oocytes were deduced according to the biopsy results and subsequent follow up data. The combined evidence suggests that one of the five oocytes started with the ring and deleted 22, two started with only the deleted chromosome 22 present and the remaining two started with the normal 22 and the deleted 22 present (Table 2 - Additional File 1).

Table 3 (Additional File 1) summarises the results of the second PGD cycle. During this cycle, 10 oocytes were collected, eight of which were normally fertilised by IVF. Seven embryos were biopsied, two cells were taken from three embryos and one cell was taken from 4 embryos with fewer than six cells. Table 4 (Additional File 1)shows the biopsy and follow up results for this cycle. No embryos normal or balanced for chromosome 22 were found at diagnosis in this cycle. Embryo 4 had the ring and deleted 22 present but the normal 22 (presumably paternal in origin) was lost in the biopsied cell.

All embryos were arrested in development at the 3–10 cell stage by day 5. Follow up results were obtained for six un-transferred embryos. Four embryos were fully chaotic i.e. with random abnormalities varying from cell to cell with no discernable mechanism. Combined diagnostic and follow up results for Embryo 5 suggest that this too originally had both the ring and deleted chromosome 22 present, but chaotic cell divisions led to the loss of either the ring or the deleted 22 in different cells. The partial monosomy 22 found at biopsy in Embryo 1 was confirmed but it was an aneuploid/chaotic mosaic by day 5. Embryo 3 appeared to be haploid with a single intact copy of chromosome 22 and a single copy of chromosome 14. The remaining embryo (number 6) had partial monosomy 22 but that was based on only one cell and could not be confirmed. The follow up results helped in the deduction of the meiotic segregation of chromosome 22 in the oocytes. It was deduced that of five oocytes two had the ring and the deleted chromosome 22 present, one oocyte had the deleted and inact chromosome 22 and another two had the ring chromosome 22 only; the latter types would lead to partial trisomy or monosomy 22 after fertilisation. In degenerate embryo number 6 there was insufficient reliable information to be able to determine the chromosomal complement of the oocyte (Table 4 - Additional File 1).

In total, 12 embryos were biopsied, and follow up information was obtained for 10 embryos. No embryos were completely normal or balanced for chromosome 22 by day 5. There was only one embryo diagnosed as balanced out of 12 biopsied and in that embryo by day 5 postzygotic errors lead to a mosaic karyotype with half the cells having lost the ring chromosome. According to the follow up studies, in all, three oocytes apparently had a balanced chromosome 22 complement but all had the deletion and the ring 22 and not the intact chromosome 22. After fertilisation all three of the embryos accumulated postzygotic errors for chromosome 22 with the end result being either diploid/aneuploid mosaic or chaotic mosaic. Embryo 3 from cycle 2 appeared to be haploid with a single intact copy of chromosome 22; re-probing for chromosomes X, Y and 18 confirmed haploidy but with a single X chromosome so that the parental origin could not be determined. The rest of the oocytes are thought to have been the unbalanced products of meiosis. Post-zygotic errors in the resulting embryos were wide ranging and very frequent in almost all the embryos. Table 5 (Additional File 1) summarises the theoretical chromosomal complement in oocytes collected during the PGD cycles and in the two natural pregnancies. Both natural ongoing pregnancies resulted from oocytes that had an extra ring chromosome (24,X,+r22) but none of the embryos from PGD cycles presented with this combination.

## Discussion

A 37 year old female carrier of a rare chromosome rearrangement was referred for PGD. She was a balanced carrier of a deleted 22 and a complementary ring chromosome 22. This is the first report of PGD for this kind of abnormality as it is extremely rare. Two cycles of PGD were carried out using FISH with case specific probes. The strategy devised for this couple was effective since the balanced carriers of the rearrangement and all unbalanced forms were identified. The position of the breakpoint within the centromere of chromosome 22 meant that by using the centromeric probe for chromosomes 14/22 in conjunction with the subtelomere probe of 14q in the second round it was possible to identify the number of centromeric signals for chromosome 22 and hence the derivative chromosomes. Unfortunately there were no embryos suitable for transfer in either cycle since none had two intact chromosomes 22. All untransferred embryos were fully analysed providing a rare glimpse of the behavior of the derivative chromosomes 22 during oogenesis and preimplantation development.

**Figure 4:**
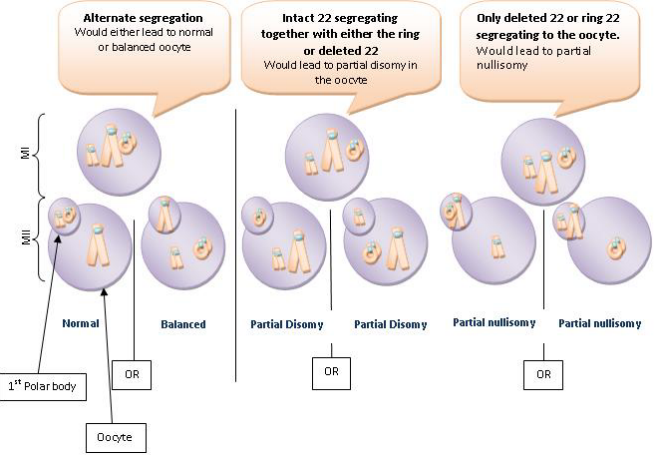
**Segregation patterns in meiosis I of oogenesis in a female carrier of del 22/ring 22 chromosomes**.

Considering the meiotic behaviour of the ring chromosome, all possible meiotic segregation patterns were seen and there does not appear to be a preferential segregation mode. Assuming that the intact chromosome 22, together with the deleted and the ring chromosome 22 were able to form a trivalent during prophase of meiosis I then there are three possible modes of segregation, only one producing balanced gametes (see Figure 4). Unbalanced products of the other two modes were all seen either in the natural pregnancies or in the embryos generated by IVF for PGD. In the case of the three embryos with presumed balanced carrier status it is assumed that the intact chromosome 22 passed to the first polar body. The evidence suggests that there is in fact random segregation in this case, presumably because normal pairing and recombination is disrupted. The reduced size of the centromeric sequences that exist in both the del(22) and the r(22) might also affect attachment to the meiotic spindle [14,15].

In one of her seminal contributions Barbara McClintock describes the mechanism leading to the formation of ring/deleted chromosomes in maize and the aberrant mitotic behaviour leading to 'variable mutant characteristics' [16]. This mechanism, a break within the centromere together with a break in either the long or the short arm, creating a small ring, has been called "centromere misdivision" [17]; these authors propose that this be referred to as "the McClintock mechanism". McClintock also describes pachytene configurations in microsporocytes, showing that although the normal, deleted and ring chromosomes may synapse, the ring is also seen with the centromeric region attached to a non-homologous bivalent. In the human situation with the ring chromosome 22, this could well be the chromosome 14 bivalent, since there is substantial centromeric homology between the two chromosomes. A rare case of a ring Y chromosome transmitted from a father to his son, who was affected by Klinefelter syndrome of paternal origin, also illustrates the effect of the ring on normal segregation of the X and Y chromosomes [18]. Additionally, several examples of maternal and paternal transmision of similar sSMC can be found in the sSMC homepage . In this case, FISH analysis of sperm from father and son showed significantly higher frequencies of diploidy and various disomies compared with control samples, suggesting that the reduced pairing efficiency of the ring Y chromosome disrupted the meiotic process generally. This is less likely to occur in the human female, since meiotic cell cycle checkpoints are less stringent than in the male [19].

Postzygotic errors were also widespread in all pre-implantation embryos studied in this case resulting in a high degree of mosaicism. The two natural conceptions also showed mosaicism. The instability of ring chromosomes is well documented in other studies both in prenatal samples and liveborn offspring[7,20-22]. The origin of this instability is partly attributed to the nature of ring chromosomes and their difficulty in undergoing mitotic division, with a tendency to form interlocking rings, leading to anaphase lag and chromosome loss [16]. In the preimplantation embryos and natural conceptions of the case studied, however, a varying degree of instability is observed in 100% of their embryonic and fetal offspring. Multiple cell lines can be seen in the preimplantation embryos due to loss of the smaller derivative chromosomes 22, or the intact chromosome, and due to chaotic cell divisions. The initial meiotic error and the instability of the r(22) and the del(22) is in addition to the frequent unbalanced mitotic divisions common in the case of preimplantation embryos. It is likely that the extreme chaotic mosaisism seen in many embryos in this case is related to the sub-fertility of the couple concerned since this type of mosaisism, although seen to be widespread during preimplantation development [23] is substantially increased in couples with repeated implantation failure [24].

In this case the ring chromosome is very stable in the mother as she is phenotypically normal and has the r(22) in all metaphases and interphases studied in her lymphocytes. The reasons for this are not well understood. One possible explanation is that the centromeric and telomeric regions required for normal cell division are still intact in the mother while in her embryos these regions may be missing or significantly shortened and their functionality reduced. Interestingly, the only well developed carrier embryo that appeared to have the same chromosomes as the mother (Embryo 2 in the first cycle) lost the ring in half of the cells by the fifth day of development, creating a mosaic with a balanced and a partially monosomic cell line. This error must have happened very early on in development possibly by the 8 cell stage or earlier, when the cell cycle checkpoints are not thought to be fully functional [25,26]. The stability of the ring in the mother could also mean that after division any abnormal cell lines are not viable and die therefore leaving only the balanced cell lines present. This cannot be proven however and the fact that the offspring in this case was mosaic and carried viable trisomic cells lines as well as the size of the small size of the imbalance (therefore viable in the trisomic or monosomic state) implies that such unbalanced cell lines might not perish.

Although the natural pregnancies of this couple both included partial trisomy 22 with mosaicism, it is expected that any of their embryos created by IVF could have produced viable unbalanced pregnancies either with partial trisomy or partial monosomy due to the small size of the chromosome involved. Chromosome imbalance of the ring 22 would be 0.6% of HAL and well within the limits of viability in the monosomic or in the trisomic state in the embryos [27].

The decision of the couple not to have the good quality balanced carrier embryo from cycle 1 transferred appears to be a valid one since the derivative chromosomes appear to be highly unstable during mitotic divisions and could produce varying abnormal phenotypes. Unfortunately, counselling couples with similar chromosomal problems is not precise. The variability of the breakpoints and the rare nature of these rearrangements as well as mosaicism and the variable phenotypes that would be produced make the task almost impossible. A similar reported case of a maternal carrier with a karyotype 47,XX,del(22)(q11q11.2),+r(22)(q10q11.2) also concluded that reproductive risks were high due to the viability of conceptions with the trisomic or monosomic states involving the ring and the deleted 22 chromosomes [28].

Considering all the above the couple concerned in this case presents with a poor prognosis in terms of producing a karyotypically normal child when all the evidence from the embryos and the previous pregnancies is taken into account. Although PGD did not produce a normal pregnancy in this case it has helped give the couple some answers concerning the nature of the reproductive difficulties they have encountered. PGD for this type of abnormality is a viable option providing that the couple is counselled that there may be no embryos suitable for transfer. The study of the preimplantation embryos in this case provided a rare and significant chance to study and understand the phenomena associated with this unusual type of anomaly during meiosis and in the earliest stages of development.

## Materials and methods

The couple was referred for PGD after the birth of a child with an abnormal karyotype initially described as 47,XY,+r(22)(p11.2q11.2)/46,XY. The child had a number of clinical features including bilateral iris coloboma, retinal coloboma, mild hypospadias and global developmental delay. After karyotypic analysis of the parents it was found that the female partner was a carrier of a balanced chromosomal rearrangement involving a ring chromosome 22 with karyotype 47,XX, del(22)(p10q12),+r(22)(q10q12). The ring is present in all her lymphocytes and is stable unlike the situation in her son where in some lymphocytes the supernumerary ring has doubled in size while in others the ring has been lost creating a normal chromosomal complement. Subsequently a second natural pregnancy with an abnormal karyotype 47,XX,+r(22)(q10.q12)[21]/46,XX[3] was terminated after chorionic villus sampling.

The couple underwent two cycles of PGD. The age of the female partner was 37 at the start of her first PGD cycle. Studies were performed on lymphocytes, single embryonic cells (blastomeres) removed from 3 day-old embryos and un-transferred embryos. Analysis was carried out using fluorescence in situ hybridisation (FISH) with specific probe sets in two rounds of hybridization. Treatment and research on embryos from this couple was carried out under licences from Human Fertilisation and Embryology Authority (HFEA) of the UK. Informed written consent was obtained for all procedures.

### Lymphocyte culture and counts

Karyotyping on all the family members had been performed by a clinical cytogenetics laboratory prior to the onset of treatment. For preparative FISH studies prior to PGD lymphocyte cultures from both parents were carried out by standard methods. Standard methods of processing and slide preparation for FISH experiments were then used [11]. The efficiency of the FISH probe combination was calculated by counting the number of correct signals in 100–200 interphase nuclei from each sample.

### IVF and stimulation protocol, embryos biopsy, blastomere and embryo spreading

Ultrasound guided vaginal oocyte collection was performed at 37 hours post hCG injection. IVF was performed at 40 and 41 hours post hCG respectively and was dependent on semen parameters and past fertilisation rates. Fertilisation was evaluated at 18–20 hours post insemination. Embryos were cultured in IVF medium (GIII series, Vitrolife, UK). On day 3, embryos that had reached at least the four cell stage were biopsied in Ca2+ -Mg2+ free biopsy medium (G-PGD, Vitrolife, UK). Two cells were removed from embryos consisting of six or more cells. Biopsied blastomeres were spread using the method described by Harper et al [29]. Cells were washed in PBS and transferred to poly-L-lysine slides in spreading solution (0.01N HCl, 0.1% Tween 20) which was gently agitated until lysis occurred and the nuclei were clear of cytoplasm. The co-ordinates of the location of the nuclei were noted using an England Finder. The same technique was used for whole embryos.

### Fluorescence In Situ Hybridisation

The combination of FISH probes used in this diagnosis was selected according to the breakpoints, firstly to detect the unbalanced products of the female meiosis for chromosome 22 and secondly to distinguish the balanced carrier embryos of the derivative chromosome 22 plus the ring chromosome from those with two intact copies of chromosome 22. For this purpose FISH probes were selected hybridizing to five different sites in two rounds of hybridisation.

**Figure 5:**
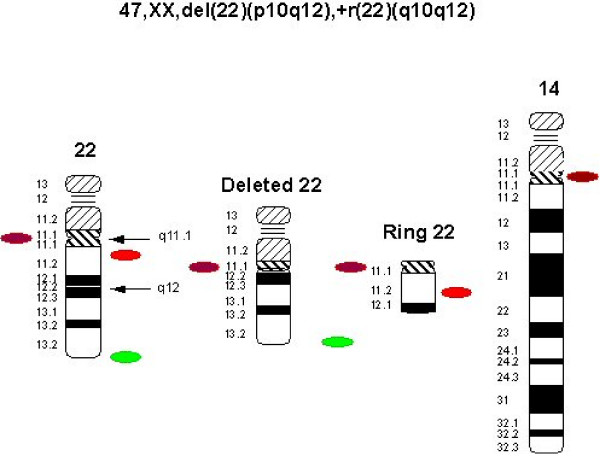
**Ideogram and probe strategy to detect the ring 22/del 22 chromosome in embryonic nuclei**. First round hybridization: DiGeorge-dual band probes (22q11.2 orange/22q13.3 green). Second round hybridization: Centromere 14/22 red and 14qtel in green (not shown).

**Figure 6:**
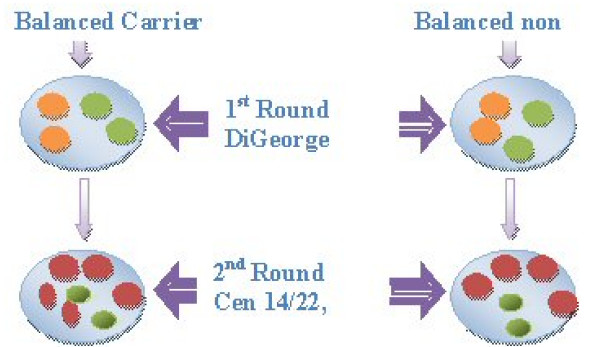
**Expected FISH signals in embryonic nuclei of balanced carriers and normal, non carriers, of the ring/deleted chromosome 22**. No difference in the number of FISH signals can be detected in the first round. While in the second round four equal sized red signals are observed for the non-carrier, the carrier presents five signals three of which are of equal size and two that are smaller. The latter combination denotes the splitting of one of the signals for chromosome 22 and thus the existence of the deleted and ring chromosomes.

In the first round the dual band LSI DiGeorge/VCFS region [Spectrum Orange (22q11.2), Spectrum Green (22q13.3)] (Abbott, UK) was used. In the second round the following probes were used: the centromeric probe for chromosome 14/22 in red (Kreatech, UK) in combination with the probe for the subtelomere of chromosome 14q in green Kreatech, UK). Figure 5 shows the karyotype of the carrier parent and the probe strategy used. The detection of carriers was possible because the chromosome 14/22 centromeric probe hybridises to both the deleted chromosome 22 and the ring 22, indicating that one of the breakpoints divides the centromeric alpha satellite sequences. However, this probe also hybridises to the centromere of chromosome 14 as well as 22 thus the subtelomeric probe for chromosome 14 had to be used in order to exclude chromosome 14 from the scoring of the chromosome 22 centromeres. Figure 6 shows the expected signals in embryonic nuclei in diploid carriers and non-carriers of the ring 22.

FISH experiments were undertaken before the PGD treatment in order to test and optimise conditions for all the probe combinations in this study. FISH for all probes was carried out using the manufacturer's instruction with minor modifications. The slides were examined under an epifluorescence Olympus microscope (Olympus BX 40) fitted with a Photometrics cooled CCD camera utilising Smartcapture software (Digital Scientific, UK). DAPI stained nuclei were located using the blue filter. Using different colour filters the scoring of signals for each of the probes to the nuclei on the slides was possible with a good degree of accuracy. All scoring decisions were made directly by viewing signals under the microscope and verified by at least two observers. Scoring criteria were applied as described in Mantzouratou *et al *[24].

## Competing interests

The authors declare that they have no competing interests.

## Authors' contributions

AM: Main author, involved in preliminary work and all data collection and analysis

MA: Data collection, involved in preliminary lymphocyte investigation

A Mania: Involved in preliminary work and biopsy data collection

SL: Main embryologist in the PGD case

PS: Main physician in the case

JD: PGD director, involved in all aspects of this case and editing the manuscript.

## Consent

Written informed consent was obtained from the patient for publication of this case report and accompanying images. A copy of the written consent is available for review by the Editor-in-Chief of this journal.

## Supplementary Material

Additional file 1 **Manuscript tables.** This file includes all tables of this manuscript. Click here for file
