# Supplementary material for: Meiotic and mitotic behaviour of a ring/deleted chromosome 22 in human embryos determined by preimplantation genetic diagnosis for a maternal carrier
Source: Mol Cytogenet. 2009 Jan 23;2:3. doi: 10.1186/1755-8166-2-3 (PMC2637284; doi:10.1186/1755-8166-2-3)
Supplement: Additional file 1 — Manuscript tables. This file includes all tables of this manuscript. [file 1755-8166-2-3-S1.doc]

| **Table 1. PGD cycle 1** |  |
| --- | --- |
| No. of oocytes collected | 7 |
| No. of oocytes fertilised | 6 |
| No. of embryos biopsied | 5 |
| No. of normal or balanced embryos on biopsy | 1 balanced carrier  (not transferred) |
| No. of unbalanced embryos on biopsy | 4 |
| Embryos with follow up result | 4 |
| Embryo progression on day 5 | 4 blastocysts  1 morula |

|  | | **Table 2. Follow up results for PGD cycle 1** | | | |
| --- | --- | --- | --- | --- | --- |
| Embryo no. | Biopsy result | | Embryo characterisation after follow up | Mechanism of  aneuploidy and mosaicism | Theoretical oocyte content for chromosome 22 |
| 1 | Partial monosomy 22: 46,--,del22(p10q12) | | Aneuploid/Chaotic mosaic embryo  Aneuploid cell line 46,--,del(22)(p10q12) | Meiotic error and chaotic cell divisions | **Del22** |
| 2 | Balanced carrier of r(22) and del(22):  47,--,del(22)(p10q12), +r(22)(q10q12) | | Balanced/aneuploid mosaic embryo.  47,--,del(22)(p10q12),+r(22)(q10q12)[7]/ 46,--,del(22)(p10q12)[7]/chaotic[3] | Loss of r(22) postzygotically | **Del22**  **R22**  **Del22** |
| 4 | Partial monosomy 22:  46,--,del(22)(p10q12) | | Aneuploid mosaic embryo  46,--,del(22)(p10q12)[10]/45,--,-22 [47] | Meiotic error and loss of del(22) postzygotically | **22** |
| 5 | Partial trisomy 22:  47,--,+del(22)(p10q12) | | Chaotic/aneuploid mosaic embryo.  47,--,+del(22)(p10q12)[5]/chaotic[9] | Meiotic error and chaotic cell divisions | **Del22** |
| 6 | Partial trisomy 22:  47,--,+del(22)(p10q12) | | No result on follow up | Meiotic error | **22**  **Del22** |

******Karyotypes based on the assumption that chromosomes other than no.22 are normal.*

|  | **Table 3. PGD cycle 2** |  |
| --- | --- | --- |
|  | No. of oocytes collected | 10 |
|  | No. of oocytes fertilised | 8 |
|  | No. of embryos biopsied | 7 |
|  | No. of normal or balanced embryos on biopsy | 0 |
|  | No. of unbalanced embryos on biopsy | 6  1 gave no result |
|  | Embryos with follow up result | 6 |
|  | Embryo progression on day 5 | All under 10 cells |

| **Table 4. Follow up results for PGD cycle 2**  ******Karyotypes based on the assumption that chromosomes other than no.22 are normal.* | | | | |
| --- | --- | --- | --- | --- |
| Embryo no. | Biopsy result | Embryo characterisation after follow up | Mechanism of aneuploidy and mosaicism | Theoretical oocyte content for chromosome 22  **R22** |
| 1 | Partial monosomy 22:  46,--,r(22)(q10q12) | Aneuploid/chaotic mosaic embryo.  46,--,r(22)(q10q12)[10]/chaotic [9] | Meiotic error and chaotic chromosome divisions | **Del22**  **22** |
| 2 | Mosaic partial trisomy 22/Partial monosomy 22  47,--,+del(22)(p10q12)/46,--, del(22)(p10q12) | Fully chaotic embryo | Meiotic error and chaotic cell divisions |  |
| 3 | Monosomy 22 and 14, intact 22 is present. | Haploid embryo  XY18 ReFISH- Only X present and one signal for 18 | One parental genome was present. | Unknown  **Del22** |
| 4 | Monosomy 22, ring and deleted 22 are present in the nuclei.  45,--,-22, del(22)(p10q12)r(22)(q10q12) | Fully chaotic embryo | Post zygotic loss of chromosome 22 and chaotic cell divisions | **R22**  **Del22** |
| 5 | Partial monosomy 22:  46,--,del(22)(p10q12) | Fully chaotic embryo with ring 22 present and deleted 22 missing | Embryo started with balanced chromosomes but chaotic cell divisions & chromosome loss followed | **R22** |
| 6 | No result | Only one nucleus with signals on follow up  Partial monosomy 22:  46,--,r(22)(q10q12) | Unknown | Inconclusive |
| 7 | Partial monosomy 22:  46,--,r(22)(q10q12) | Fully chaotic embryo | Meiotic error and chaotic cell divisions | **R22** |

| **Table 5. Deduced Meiotic Segregation in oocytes**  **22**  **22**  **22** | | | | | | |
| --- | --- | --- | --- | --- | --- | --- |
| **Theoretical meiotic segregation in oocyte** |  | **R22**  **Del22** | **Del22** | **R22** | **Del22** | **R22** |
| **Cycle no.1** | 0 | 1 | 2 | 0 | 2 | 0 |
| **Cycle no.2** | 0 | 2 | 0 | 2 | 1 | 0 |
| **Natural pregnancies** | 0 | 0 | 0 | 0 | 0 | 2 |
| **Total** | 0 | 3 | 2 | 2 | 3 | 2 |
